# The development of a high-density genetic map significantly improves the quality of reference genome assemblies for rose

**DOI:** 10.1038/s41598-019-42428-y

**Published:** 2019-04-12

**Authors:** Shubin Li, Guoqian Yang, Shuhua Yang, Jeremy Just, Huijun Yan, Ningning Zhou, Hongying Jian, Qigang Wang, Min Chen, Xianqin Qiu, Hao Zhang, Xue Dong, Xiaodong Jiang, Yibo Sun, Micai Zhong, Mohammed Bendahmane, Guogui Ning, Hong Ge, Jin-Yong Hu, Kaixue Tang

**Affiliations:** 1 National Engineering Research Center For Ornamental Horticulture, Flower Research Institute, Yunnan Academy of Agricultural Sciences; Yunnan Flower Breeding Key Lab, Kunming, 650231 China; 2 CAS Key Laboratory for Plant Diversity and Biogeography of East Asia, Kunming Institute of Botany, Chinese Academy of Sciences, Kunming, 650201 China; 3 Institute of Vegetables and Flowers, Chinese Academy of Agricultural Sciences, Beijing, 100081 China; 4 Laboratoire Reproduction et Développement des Plantes, Univ Lyon, ENS de Lyon, UCB Lyon 1, CNRS, INRA, F-69364 Lyon, France; 5 Key laboratory of Horticultural Plant Biology, Ministry of Education, College of Horticulture & Forestry Sciences, Huazhong Agricultural University, Wuhan, 430070 China; 6 Kunming College of Life Sciences, University of Chinese Academy of Sciences, Kunming, 650201 Yunnan Province China; 7 Germplasm Bank of Wild Species, Kunming Institute of Botany, Chinese Academy of Sciences, Kunming, 650201 China

## Abstract

Roses are important woody plants featuring a set of important traits that cannot be investigated in traditional model plants. Here, we used the restriction-site associated DNA sequencing (RAD-seq) technology to develop a high-density linkage map of the backcross progeny (BC1F1) between *Rosa chinensis* ‘Old Blush’ (OB) and *R. wichuraiana* ‘Basyes’ Thornless’ (BT). We obtained 643.63 million pair-end reads and identified 139,834 polymorphic tags that were distributed uniformly in the rose genome. 2,213 reliable markers were assigned to seven linkage groups (LGs). The length of the genetic map was 1,027.425 cM in total with a mean distance of 0.96 cM per marker locus. This new linkage map allowed anchoring an extra of 1.21/23.14 Mb (12.18/44.52%) of the unassembled OB scaffolds to the seven reference pseudo-chromosomes, thus significantly improved the quality of assembly of OB reference genome. We demonstrate that, while this new linkage map shares high collinearity level with strawberry genome, it also features two chromosomal rearrangements, indicating its usefulness as a resource for understanding the evolutionary scenario among Rosaceae genomes. Together with the newly released genome sequences for OB, this linkage map will facilitate the identification of genetic components underpinning key agricultural and biological traits, hence should greatly advance the studies and breeding efforts of rose.

## Introduction

Rose (*Rosa sp*., Rosaceae) is one of the most important horticultural plants. Besides its high ornamental values as garden plant and cut flowers, it also provides key materials for production of essential oils used in perfume and cosmetic products, and for food and medical products^1,2^. Roses have been cultivated since antiquity but the breeding activity that led to the production of modern roses only really began in the 19^th^ century^3^. In the genus of *Rosa*, there are about 200 species, among which 95 species are found in China, and more than 35,000 commercial cultivars (http://www.efloras.org). Most of the modern roses have a long and complex history of hybridization/crossing and polyploidization process among a dozen species^4–7^. Due to high frequency of backcrossing with Asian germplasms, cultivated roses display a shifting pattern from European to Asian genetic backgrounds^8,9^. Besides their economical importance, roses feature key biological traits such as scent production, continuous flowering (CF) and double flowers. Rose is now becoming a model species for woody plants as it has a relatively small genome (approximately 560 Mb) whose sequence was very recently released^6,10^ and an established transgenic systems^7,11–14^.

The market for high-quality roses demands the continuous development of new varieties with better performance, such as disease resistance and flower quality. Rose breeding often requires tedious crosses between species, while marker-assisted selection (MAS) holds great promise for faster breeding of rose cultivars by speeding up progeny screening^15,16^. A high-resolution genetic map could be useful for map based cloning of genes associated with traits of interest^17^, and to improve genome assembly and comparative genomics^18^. It will provide an opportunity to understand the genomic architecture and chromosomal rearrangements that occurred during *Rosa* speciation and domestication.

The first molecular genetic linkage map for rose was constructed mostly with RAPD and AFLP markers by using a diploid F1 population derived from *Rosa multiflora* hybrids with the double pseudo testcross strategy^19^. Since then, several genetic maps have been constructed for diploid and tetraploid roses using AFLP, RFLP, SSR and/or CAPS markers^20–27^. Meanwhile, several important traits like flower color^19^, presence of prickles on the stem^19,24^, resistance to black-spot^28,29^ and powdery mildew^30^, and flowering time^25,27^, have been mapped on these genetic maps^23,31^. However, in roses, the low marker density, along with the high costs and the time and labor required to develop new markers, make most of these maps unsuitable for fine mapping of traits of interest and useless for breeding programs. Whole genome-wide DNA markers are required to construct high-resolution linkage maps. One important type of DNA markers is SNP (single nucleotide polymorphisms). Recently, one 68k EST-based WagRhSNP array was developed for roses and allowed expanding existing tetraploid rose maps^10,32,33^.

Next-generation sequencing (NGS) technology provides opportunities to genotype a large number of individuals and unravel large number of SNP markers at the same time without the need of a reference genome sequence^34,35^. Two typical methods to develop genome-wide markers with NGS technology are genotyping by sequencing^36^ and restriction site-associated DNA sequencing (RAD-seq)^37–39^. RAD-seq utilizes the power of NGS platforms to generate high coverage of short tags adjacent to restriction sites, which can then be used to identify single nucleotide polymorphisms (SNPs) between genotypes. Recently, SNP-based consensus-maps were constructed by RAD-seq for diploid roses^10,40^. Though genotyping by sequencing has been proved to be successful in detection of bi-allelic SNP markers in both diploid and tetraploid roses, a high-density genetic map for founder species of modern roses remains necessary to facilitate QTL positioning, map based cloning and comparative genomics studies^10,40,41^.

*Rosa chinensis* ‘Old Blush’ (OB) and *R. wichuraiana* ‘Basye’s Thornless’ (BT) are among the founder genotypes during rose domestication. OB contributed several novel but important traits such as CF and tea scent^7,8,42–46^. OB and BT feature several contrast phenotypes like continuous flowering (CF), number of petals, etc^42^. Previously, the development of a backcross population between OB and BT (BC1F1) has allowed us to genetically identify loci controlling recessive traits, like CF trait^42^, while the comparisons between OB and BT transcriptomes together with other Rosacaeae plants provided us the opportunity to identify molecular features characterizing roses^47^. In this study, we used RAD-seq to screen for SNP markers in this population. This allowed us to construct a high-density SNP-based genetic map. Our map comprised seven linkage groups with a total of 2213 high quality SNP markers and was integrated with the current genome assemblies to define the scaffolds order.

## Results

### RAD-seq library construction and sequencing

A total of four RAD-seq libraries from the two parents and their 152 offsprings were constructed and sequenced. Approximately 6.55 Gb (BT) and 7.24 Gb (OB) raw data were generated prior to any quality filtering for the parents, while 6.52 Gb (BT; 21.73 million reads) and 7.22 Gb (OB; 24.09 million reads) were kept after quality filtering (Supplemental Table S1). About 95.6% (minimum 93.38%) of reads had an average quality higher than Q20 (Q20 indicates a 1% chance of error), indicating the high-quality of the data. For the 152 individuals, sequencing of the RAD libraries generated a total of 193.12 Gb raw data. Of the raw reads, an average of 3.55 million reads per individual were retained after removing the putatively duplicated reads and reads without intact *Eco*RI cutting sites (average complete enzyme digestion ratio was 94.32%). After quality filtering, a total of 191.69 Gb clean data (99.23%) were retained with an average of 1.07 Gb per individual, which ranged from 0.49 to 4.97 Gb (Supplemental Table S1; Fig. S1). Overall, our RAD-seq data showed a high Phred quality (Q20 ≥ 93.38%, Q30 ≥ 86.76%), a stable GC content ranging from 36.81% to 38.41% and a high digestion rate from 85.94% to 97. 80%.

### SNP discovery and genotyping

The number of RAD tags detected in the male parent (BT) and female parent (OB) was 296,621 and 198,349, with an average depth (the average number of reads per tag) of 58.35 and 78.69 times, respectively (Supplemental Table S2). For the progeny individuals, the number of tags ranged from 110,760 to 243,497 with an average of 167,392 and a mean depth of 16.08 times (8.29 to 181.53 times) (Supplemental Table S2, Fig. S2). After merging alleles together, 357,174 tags were detected, among which 139,834 were polymorphic with a polymorphism rate of 39.15%. A total of 593,497 SNPs were identified in the polymorphic tags with an average of 4 SNPs in each tag. These SNPs were then classified into transitions and transversions based on nucleotide substitution. Transitions (59.37%) were the main type and about 1.46 times larger than the transversions type (40.62%). The number of A/G transitions was nearly equal to C/T transitions; the numbers of A/C, A/T, and G/T transversions were nearly equal, however, almost two times higher than C/G transversions (Table 1).

**Table 1:** Types of SNPs (transition/transversion) for double alleles loci.

Transition	Counts	Transversion	Counts
G-A	175644 (29.59%)	A-T	75808 (12.77%)
T-C	176740 (29.78%)	A-C	62386 (10.51%)
		G-T	64681 (10.90%)
		G-C	38238 (6.44%)

### High-resolution genetic map construction

**Figure 1:**
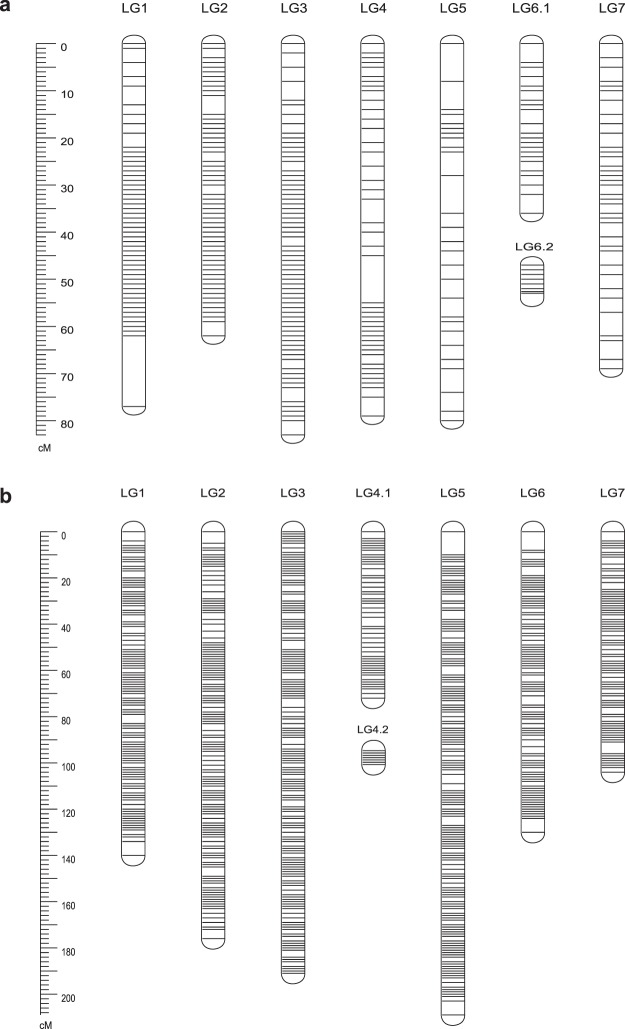
Linkage group length and marker distribution on the Version 1.0 genetic map (**a**) and Version 2.0 genetic map (**b**) of rose. Genetic map details are provided in Supplementary Table S2.

We used a two-step strategy to construct the genetic map. First, a screen for markers that were present in at least 140 individuals (missing data ratio <6%) identified a total of 793 markers. *JoinMap 4.0* and *Lep-Map 2.0* softwares were used to build linkage groups after removing 277 markers that were severely distorted (χ^2^ > 30, P < 0.01). Three individuals were excluded during marker analysis and mapping due to excessive missing alleles. Obscure markers that were assigned to different locations of a linkage group were removed after comparing the marker location determined by the two programs, and then the genotype matrix of rest of the markers were re-ordered until the relative order between markers arranged by the two programs did not conflict. This map was designated as Version 1.0 (Table 2, Fig. 1a). The Version 1.0 genetic map spanned a total of 492.3 cM with a total of 552 markers distributed on 7 linkage groups (LG6 was split into two parts), consistent with the haploid chromosome number (n = 7). Overall, each linkage group contained 79 markers that spanned an average length of 70.3 cM, with a mean marker interval of 1.3 cM. The number of mapped markers per linkage group varied from 22 markers on LG6 to 188 markers on LG1. The smallest linkage group was LG6, which contained 71 markers spanning a length of 42.52 cM. The largest linkage group was LG4, which had 100 markers and a length of 85.81 cM. The maximum gap size in each linkage group ranged from 1.00 cM on LG6 to 14.86 cM on LG1, with an average of 6.33 cM (Table 2).

**Table 2:** Key statistics for the linkage groups (LGs) of Version 1.0 and Version 2.0 maps.

LGs	Nb. of markers	map_size (cM)	average gap_size (cM)	biggest gap_size (cM)	Nb. Of unique positions
**Version 1.0**					
LG1	188	77.132	0.41	14.863	188
LG2	79	61.572	0.79	3.948	79
LG3	97	83.007	0.86	4.117	97
LG4	51	79.18	1.58	9.696	51
LG5	27	80.221	3.09	8.071	27
LG6.1	22	35.885	1.71	4.174	22
LG6.2	49	6.631	0.14	1.001	48
LG7	39	68.676	1.81	4.808	39
all	552	492.304	1.29875	14.863	551
**Version 2.0**					
LG1	386	140.202	0.97	6.077	145
LG2	325	176.381	1.12	5.008	158
LG3	393	190.96	1.01	3.792	191
LG4.1	114	71.889	1.22	4.521	60
LG4.2	53	5.836	0.11	0.534	53
LG5	503	208.63	1.07	9.953	196
LG6	243	129.579	1.07	7.969	122
LG7	196	103.948	1.08	5.623	97
all	2213	1027.425	0.96	9.953	1022

To increase the number of markers on the genetic map, we next decreased the stringency and screened for markers present in at least 122 individuals (missing data ratio <18%). A total of 3145 markers were retained. After removing 903 markers with severe distortion (χ^2^ > 30, P < 0.01) and 4 individuals with an excessive of missing alleles, *Lep-Map 2.0* was used to construct the Version 2.0 of the rose genetic map. To refine Version 2.0 map, we compared it to Version 1.0. After removing markers with inconsistent orders, we recalculated the genotype file and obtained the final rose map Version 2.0 (Table 2, Fig. 1b). Version 2.0 map showed a high consistent order of markers with the Version 1.0 except for some outliers (Supplemental Figs S3, S4).

The total length of Version 2.0 was 1027.4 cM, with a total of 2213 markers (1022 unique positions). The average distance between adjacent markers was 0.96 cM. The number of markers mapped per LG ranged from 167 (LG4) to 503 (LG5), with an average of 316. Linkage groups vary widely in size, with an average length of 146.77 cM. The largest linkage group was LG5, with a length of 208.63 cM and a mean marker interval of 1.07 cM, while the smallest linkage group was LG4, with a length of 77.72 cM and an average distance of 0.687 cM between adjacent markers. The maximum gap size in each linkage group ranged from 0.53 cM on LG4 to 9.95 cM on LG5 (Table 2). The LG4 linkage group was short and broken into two parts (Fig. 1b), which could be related to the fact that the middle part of LG4 is highly enriched for repeat sequences, and thus a low occurrence of restriction enzyme cutting sites was found. Location of clustered markers, some of which could associate with the centromeric regions, was prominent on linkage groups LG1, LG2, LG3, LG5, LG6, and LG7 (Supplementary Fig. S5). Marker names and positions for all SNP loci in the seven LGs of the genetic map were listed in Supplementary Table S4.

### Distribution of distorted markers on the genetic map

**Figure 2:**
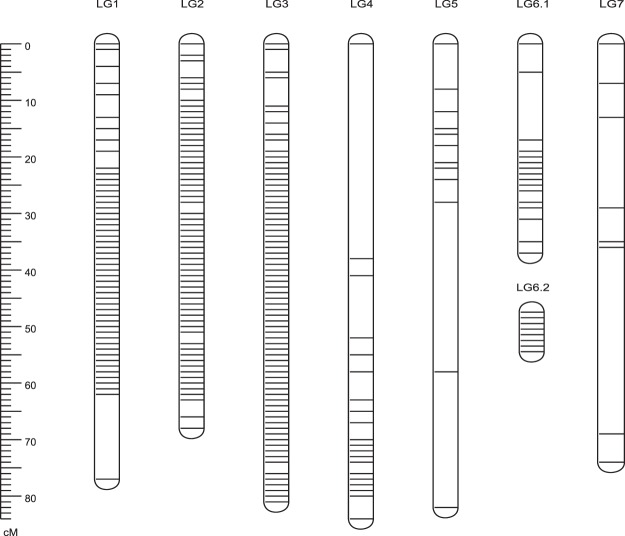
Distribution of distorted markers on the Version 1.0 rose genetic map.

In the Version 1.0 map, after the Chi-square test of genotypes, 607 markers (76.54%, P < 0.01) severely deviated from the 1:1 segregation ratio, while 61 markers (7.69%, 0.05 < P < 0.01) deviated mildly from the 1:1 segregation ratio (a total account of 84.2% markers). In the Version 2.0 map, 2187 markers (69.54%, P < 0.01) were found to be severely distorted from the 1:1 allele frequency, and 206 markers (6.55%, 0.01 < P < 0.05) were mildly deviated from 1:1 segregation after Chi-square test of genotypes, accounting for 76.09% of the total number of markers. We added distorted markers on the final map only when they did not affect the original orders of marker. To examine distribution of the distorted markers, we mapped all of them on the Version 1.0 map (Fig. 2). These markers were unevenly distributed along the seven LGs and tended to form 11 blocks of segregating distortion (RSD) on 6 LGs including 557 markers (Fig. 2; Supplementary Table S5). The smallest RSD block contained 8 markers (1.7 cM), while the largest one contained 162 markers (81.19 cM). LG1 contained the most RSDs, while LG7 contained the least distorted markers and no RSD was found on LG7. Interestingly, the seven potential self-incompatibility-related genes (three encoding for S RNAses and four encoding S-locus type F-box proteins) were all found on the first RSD of LG1 (17.14~23.59 cM, Chr3: 40570 Kb~40670 Kb)^10^. There were 144 distorted markers caused by an excess of heterozygous alleles (20.77%, χ^2^ test, P < 0.05), while the other 549 markers were due to excess of homozygous alleles (79.22%, χ^2^ test, P < 0.05) (Supplementary Fig. S6).

### Evaluation of the genetic map

**Figure 3:**
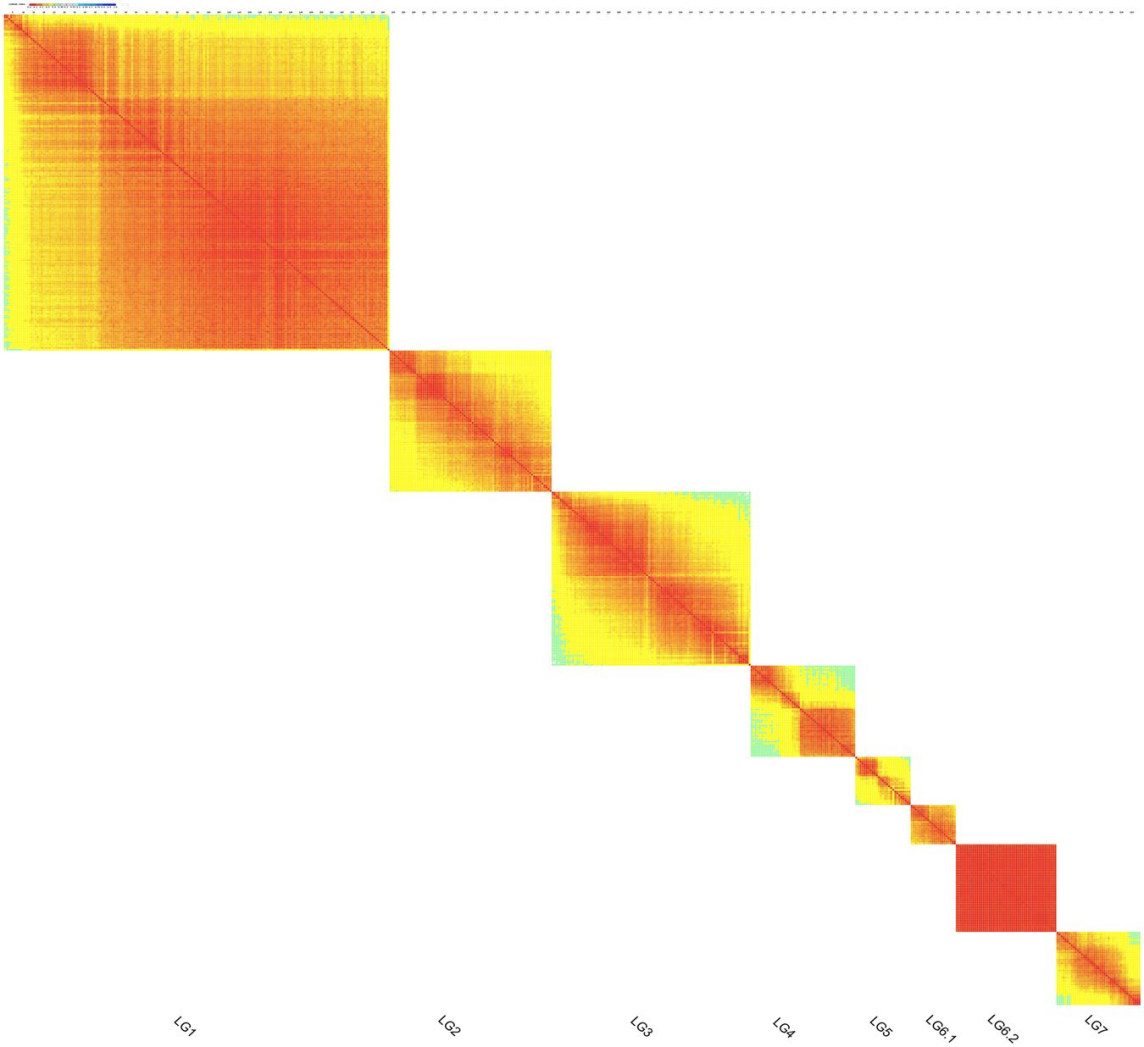
Heat maps reflecting the recombination relationship between markers in each linkage group. Each cell represents the recombination rate of two markers. Yellow color indicates a lower recombination rate while red color indicates higher recombination rate.

We evaluated the Version 1.0 map with heat map (representing the recombination relationship between markers in each LG and identification of potential ordering errors) and with haplotype map (graphical genotypes, detecting the occurrence of double crossover events) assays. Heat maps showed that most of the 552 mapped-markers in most LGs were well ordered (Fig. 3). Consequently, graphical genotypes were generated based on these markers (Supplementary Fig. S7). From the color change indicates the occurrence of a recombination event we defined the majority of recombination blocks and identified relatively low proportion of double crossover and missing markers for each LG. Among the 754,284 contigs produced via clustering the 6 Kb library reads, seven pairs of adjacent markers that could hit the same contig were detected, evidencing the reliable order of these markers (Supplementary Table S6, Fig. S8). The final map covered more than 99.12% of the diploid rose genome.

### Relationships between genetic and physical maps

**Figure 4:**
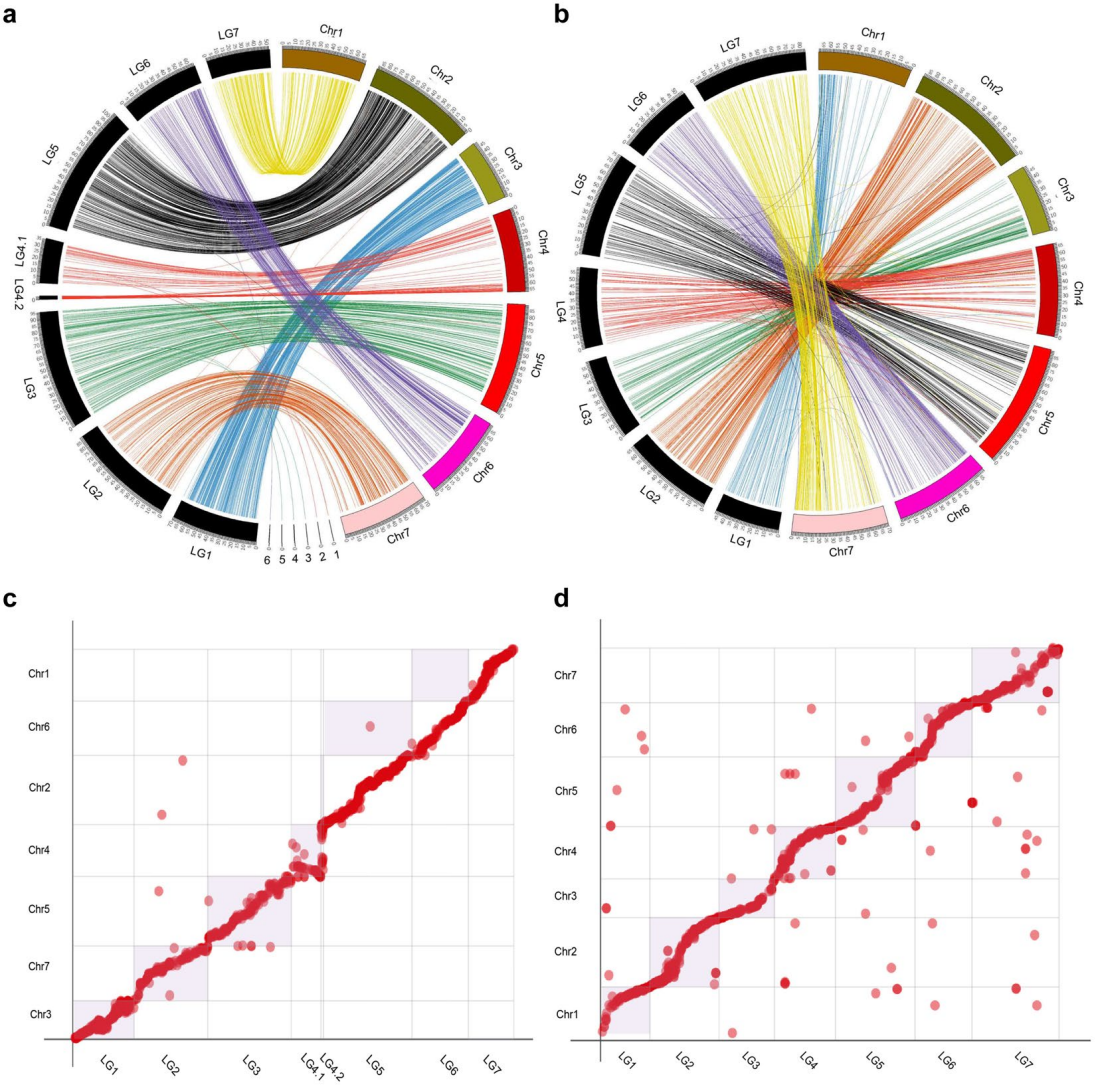
High conservation level of synteny between the constructed genetic map and the OB reference chromosomes^6^. (**a**,**c**) Comparison of the syntenic relationship between the seven LGs (LG1-7) constructed in this research (in black) with the seven published OB reference Chromosomes (Chr1-7, in brown). 1–6 marks the unassigned and unordered contigs in OB reference genome. (**b**,**d**) Comparison of the syntenic relationship between seven LGs published by Yan *et al*.^40^ (in black) with the reference OB genomes (in brown). Chromosome 0–7 marks the published OB assembly.

To determine the relationships between our genetic map and the newly published physical map^6^, marker sequences from Version 2.0 map were aligned to the OB genome. The OB doubled-haploid genome contains seven pseudomolecules that were built by integrating 25,695 markers of the K5 rose high-density genetic map. Using BLAST searches, a total of 1,371 RAD markers showed a unique match to the *R. chinensis* genome. Notably, most of the 7 linkage groups were syntenic with one of the 7 chromosomal pseudomolecules from the assembly, while only <0.5% markers mapped onto linkage groups conflicted with their position in the assembled chromosomes (Fig. 4a). Chromosome segments with inversion or rearrangement were found at the end of LG1 and LG3. The discrepancy may be due to miscalculation, which occurs usually at both ends of a linkage group during linkage analysis. To clarify this situation, we compared the rose GBS genetic map published by Yan *et al*.^40^ with the published rose genome and detected similar rearrangements at the end of LG4, LG5, LG6 and LG7 (Fig. 4b). Furthermore, we observed a chromosome-segment-inversion between the middle of LG1 (30–40 cM) and the Chr3 of the genome. This inversion is still presence even if we use *JoinMap* to reorder this LG, indicating that it is not caused by random error during map construction. As the genome assembly is of high-quality and confirmed by mapping of Hi-C chromosomal-contact-map data^6^, this discrepancy might be due to genomic rearrangements between OB and BT, but not to scaffold misplacement which were usually found in some genome sequencing projects. The highly heterozygous OB genome could provide an alternative explanation for this discrepancy. However, we cannot exclude the possibility of genotyping errors due to low sequencing depth of some markers.

The RAD-seq based genetic map was further used to improve the OB genome assemblies^6,10^ (here denoted as the Raymond’s and Hibrand Saint-Oyant’s genomes, respectively). For the Raymond’s genome, six additional previously unrecorded contigs (1.21 Mb) were anchored to specific chromosomes, accounting for 12.18% of the unanchored contigs (9.94 Mb). However, we were unable to determine their orientation on the chromosome because each contig contained only one marker. The list of newly anchored contigs to the LGs is presented in Table 3. We also identified 11 contigs containing a significant amount of centromeric repeats (Table 3, Supplementary Table S8). *Circos* plot of 7 pseudo-chromosomes with genetic (in cM) to physical (in Mb) distances is presented in Fig. 4. For the Hibrand Saint-Oyant’s genome, about 1240 markers were anchored to the seven chromosomes, while about 103 hit to the unassigned contigs (~44.52%, 23.14 Mb of the 52 Mb Chr0). Among all the unassigned contigs, one contig was of chloroplast origin, eight were from mitochondria, and 231 were centromeric (Supplementary Table S9). No marker hits to the centromeric contigs. In general, these results reveal the potential of this map in improving rose genome assemblies (Supplementary Fig. S9; Table S7).

**Table 3:** List of the newly anchored scaffolds to the Raymond’s genome^6^.

LG	Marker	Location (cM)	Scaffold	Location (bp)
LG3	98870	68.563	Chr0c37 (NW_020126846.1)	61,300
LG3	200853	99.204	Chr0c11 (NW_020126825.1)	44,754
LG3	200854	99.866	Chr0c21 (NW_020126834.1)	132,452
LG4.1	192439	15.729	Chr0c45 (NW_020126852.1)	33,995
LG4.1	192439	15.729	Chr0c45 (NW_020126852.1)	93,608
LG4.1	51116	29.972	Chr0c16 (NW_020126829.1)	69,353
LG4.1	51116	29.972	Chr0c16 (NW_020126829.1)	45,922
LG6	234097	30.062	Chr0c15 (NW_020126828.1)	213,124
Centromere			Chr0c43	
Centromere			Chr0c24 (NW_020126837.1)	
Centromere			Chr0c31 (NW_020126842.1)	
Centromere			Chr0c08 (NW_020126822.1)	
Centromere			Chr0c02 (NW_020126816.1)	
Centromere			Chr0c18 (NW_020126831.1)	
Centromere			Chr0c12 (NW_020126826.1)	
Centromere			Chr0c14	
Centromere			Chr0c46	
Centromere			Chr0c42	
Centromere			Chr0c25 (NW_020126838.1)	

### Synteny between rose and *Fragaria vesca*

**Figure 5:**
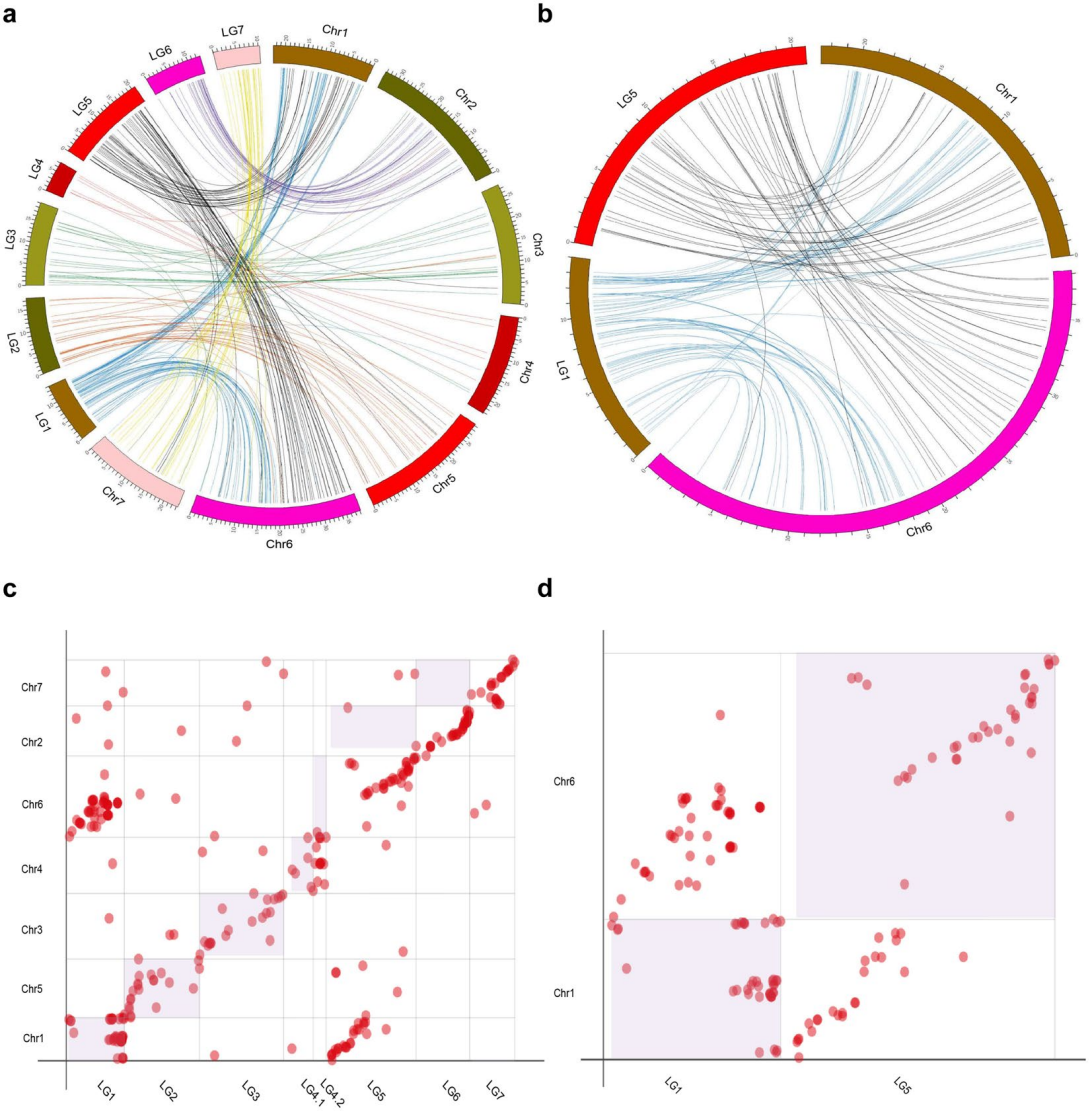
Comparison of the newly constructed linkage map and *Fragaria vesca* genome. (**a**,**c**) Comparison of the syntenic relationship between the LGs constructed in this study (LG1-7) with the seven *F. vesca* chromosomes (v1.1; Chr1-7). (**b**,**d**) Detailed comparison of the syntenic relationship between LG1 and LG5 with *F. vesca* Chr1 and Chr6 showing chromosomal rearrangements.

*Rosa sp*. and *Fragaria vesca* (wild strawberry) exhibit high synteny^6^. Thus we investigated the correspondence between our newly constructed LGs and *F. vesca* chromosomes. About 13.75% (297) tags on our genetic map have homologs in *F. vesca* genome (v1.1 assembly). Among the 297 homologs, 287 were in highly syntenic regions and collinear with *F. vesca* chromosomal regions. An apparent 1:1 correspondence relationship between five *F. vesca* chromosomes (2, 3, 4, 5, and 7) and five of our rose LGs (2, 3, 4, 6 and 7) was observed with a good collinearity along the entire LGs. The remaining two LGs (1 and 5) showed chromosomal rearrangements (fusions and fissions) (Fig. 5, Supplementary Table S10). Rose LG1 shares synteny with half of Chr1 and half of Chr6 of strawberry, while LG5 features synteny with the remaining segments of strawberry Chr1 and Chr6 (Fig. 5). A further comparison with the newest version of *F. vesca* genome assembly (v4.0.a1)^48^ showed the same patterns (Supplementary Fig. 10). These differnces could be due to inter-chromosomal rearrangements after the divergence of the *Fragaria* and *Rosa* species from their last common ancestor. Instead, a comparative genomic study within the Rosaceae family showed that rose and strawberry originate from a common ancestral Rosoideae Karyotype (ARoK) through a chromosomes fusion for strawberry genome, whereas the rose went through one fission and two fusions, independently from strawberry^6^. Our pattern is thus consistent with these published paleogenomic results and also with other data based on the linkage map of diploid and tetraploid roses as well as the rose whole genome assemblies^6,10,32,40^.

## Discussion

In this study, we used RAD-seq technology to construct a high-density linkage map with 139,834 polymorphic SNP tags and 2,213 markers between two rose genotypes and their BC1F1 progeny. The new linkage map contains seven LGs with a total length of 1,027.4 cM and an average marker spacing of 0.96 cM. While all previously published maps were based on F1 mapping populations utilizing pseudo-testcross mapping strategy, this study represents the first rose map constructed by using RAD-seq on a BC1F1 population. This newly constructed map will be highly valuable for dissecting the molecular genetics of important trait, especially those with recessive inheritance nature^42^.

This map possesses two major advantages over previous maps. First, the map is of high density and high resolution. The 2,213 markers (1,022 unique positions) cover about 1,027.4 cM on the seven LGs with an average marker density of 2.15 markers per cM. Therefore, this map represents an improvement over the integrated consensus map based on SSR and AFLP markers reported previously^31^, and offers rose geneticists and breeders a broader suite of options of marker selection for a wide range of research purposes. Secondly, all markers in this map are sequence-defined, and can be easily and unambiguously identified in any rose germplasm. These are very useful for comparative genomic studies with other plant species. It’s worthy to note that markers in this map are not uniformly distributed on each LG (Fig. 1, Supplementary Fig. S5). We observed clustering of markers into short intervals in almost all LGs, which could be due to suppressed recombination at the telomeric and centromeric regions^49,50^. However, this clustering may also reflect the unequal distribution of *EcoR*I cutting sites in different chromosomal regions^24^. Despite the high-density of this RAD-seq based map, gaps still exist in most of the LGs. Indeed, there are more than 10 gaps exceeding 5 cM with the largest gap covering 9.953 cM on LG5. Identifying additional markers with a different enzyme or enzyme pairs (for example, *Ava*II + *Msp*I)^39^ should help filling these large gaps.

We used a two-step strategy to generate this map for guaranteeing the quality and reliability. Parental linkage groups were first constructed using high quality markers with low missing ratio using simultaneously the JoinMap and Lep-Map programs with high LOD score (LOD ≥ 6.0). Different algorithms for ordering markers in the two programs corroborated the accuracy of our rose genetic map Version 1.0. Next, we added, to this Version 1.0, markers with high missing ratio, while excluding those that may cause conflicting marker orders. The final map, resulting from several rounds of grouping, is more reliable than maps generated with genotypes of high missing ratio because of the presence of genotyping errors in markers and wrong estimates of recombination rates (Supplementary Fig. S4). Since both heat map and haplotype map confirmed that most markers were well ordered in all LGs, and the order of most of these markers on the map was consistent with that observed in the reference genome^6^, we conclude that the newly developed integrated map is of high quality and reliability. Additionally, this newly constructed map is rather complete, though more markers are necessary to reinforce, for example LG4.1 and LG 4.2.

High frequency of segregation distortion (SD) is not unusual in outcrossing Rosaceae species^50,51^ like roses^24,26^. In *Fragaria*, 54% of the polymorphic loci deviated from the expected 3:1 or 1:2:1 ratio in a F2 population. In rose F1 populations, it was observed that 28.9% of the markers in H190 (male parent) deviated significantly from the expected segregation ratio of 1:1, while 50% of the markers in *R. wichuraiana* (female parent) deviated significantly from the expected segregation of 1:1 ratios at P = 0.005^24^. In the 93/1-119 (P119) X 93/1-117 (P117) F1 population, about 22% of distorted markers were found (P ≤ 0.05)^26^. As for our newly constructed map, 84.2% markers departed from the expected 1:1 Mendelian segregation ratio in the Version 1.0 map, and 76.09% in Version 2.0 map (χ^2^, 0.05 < P < 0.01). This pattern has ever been detected in a pummelo (*Citrus grandis*) F1 population^52^. One possible explanation could be that segregation distortion was brought in by non-randomized sampling and data filtering as well as insufficient sequencing depth. Insufficient sequencing could produce over-representation of one of the alles at the heterozygous locus and thus cause segregation distortion. At the same time, we cannot rule out the possibility that certain genotypes remain undetected because of incomplete digestion by the restriction enzyme. Another explanation can be that biological factors, e.g. gametophytic selection and lethal genes, could also cause segregation distortions^24,26,51^. Gametophytic selection by sub-lethal genes conferring “low viability” of zygote, embryo or seedling, could be located close to the distorted markers in maps. Presence of a self-incompatibility locus in the 93/1-119 (P119) x 93/1-117 (P117) F1 population and in *R. rugosa* has been reported^26,53^. 557 markers in our map were unevenly distributed along the seven LGs and formed 11 regions of segregating distortion (RSD) on 6 LGs including. Indeed, one RSD region harbored the potential self-incompatible loci (RNases and F-box proteins)^10^. However, the high level of synteny between our genetic maps and chromosomal-level physical maps^6,10^ indicates that the high proportion of distorted markers is more likely due to biological factors (genetic divergence between the parent plants) than to technical bias in the genotyping process.

Chromosome inversions were detected in rose genomes. Though our newly constructed map featured a good collinearity with the reference genome assembly, an inversion in the middle of LG1 (Chr3) was observed using both *JoinMap* and *Lep-Map* approaches. This inversion is not present in a rose consensus map (J06-20-14-3×‘Little Chief’, J06-20-14-3×‘Vineyard Song’ and ‘Old Blush’×‘Red Fairy’)^40^. Distorted segregation of chromosome rearrangement among different species might explain this phenomenon^54^. Further studies with cytogenetic methods and a bigger mapping population should clarify this situation.

Genome architecture varies in roses. The presented genetic map revealed a high level of synteny conservation and genome rearrangements during evolution of roses and strawberry from their last common ancestor(s). The ancestral karyotype of Rosoideae (including strawberry and rose, RoAP) had eight protochromosomes^6^. Strawberry may have experienced an ancestral chromosome fusion from the eight protochromosomes to reach its current genome structure, while rose genome went through one independent fission and two fusions^6^. Both rose LG1 and LG5 are formed by a fusion of strawberry Chr1 + Chr6 fragments (Fig. 5). This is consistent with observations from whole genome sequencing and genetic maps of the diploid and tetraploid roses^6,10,32,33,40^. The potential breakpoints are close to predicted centromere positions, thus fits well with a telomere-centric model underlying karyotype evolution of most plants^55^. Genome diversity observed in this newly constructed genetic map and known maps as well as in the reference genome sequences should provide important information on genome evolution in Rosaceae.

The newly constructed map and the available reference genome sequences will greatly promote roses to become models for studying flowering behavior variation and other important traits in ornamental plants^13^. Indeed, this genetic map significantly improves the quality of reference genome assemblies. Thanks to the markers of this map, about 1.21 Mb (12.18%) and 23.14 Mb (44.52%) of the unassigned contigs could be anchored to the two recently published reference rose genomes generated from doubled-haploid OB plants^6,10^. As we excluded the markers with multiple hits to the unassigned contigs, no marker associated with unassigned centromeric-contigs was detected. Integration of this high-density linkage map with the assembled genomes should benefit significantly the dissection of molecular mechanisms underpinning key biological traits and thus improve the breeding of roses^42^.

## Materials and Methods

### Plant materials and DNA extraction

The BC1F1 population between *Rosa chinensis* ‘Old Blush’ (OB) and *R.* *wichuraiana* ‘Basyes’ Thornless’ (BT) was developed as described previously^42^. Briefly, a F1 progeny was produced via crossing the vegetatively propagated OB and BT plants. Then the F1 plants with simple flowers (five petals only) were taken as the female plants and pollinated by pollens from OB. This process was repeated in 2013 and 2014 to generate a population of 152 BC1F1 individuals. This population segregates for at least six pairs of biological traits including continuous flowering and petal numbers. For genotyping and RAD-seq, young healthy leaves from two parents and BC1F1 individuals were collected. Genomic DNA was extracted with CTAB method^56^ and quantified with a NanoDrop ND1000 spectrophotometer and Qubit 2.0 Fluorometer (Thermo Fisher Scientific, Rochester, NY, USA). DNA concentrations were adjusted to 50 ng/µl using Tris-EDTA buffer.

### RAD library construction and high-throughput sequencing

RAD library construction, sample indexing and pooling were carried out as previously described^37^. DNA of two parents and each of their derived offspring was digested with *EcoR* I (New England Biolabs, Ipswich, MA, USA). Various P1 adapters, each with a unique 4–8 bp molecular-identifying sequence (MID, or barcode), were then ligated to designated individuals with T4 DNA ligase (New England Biolabs, Ipswich, MA, USA). The adapter-ligated products were then pooled in groups of 24 individuals and randomly sheared into DNA fragments. Sheared DNA was purified, eluted, and separated using gel electrophoresis, and DNA bands corresponding to 300–700 bp were excised and purified with magnetic beads. After end repair, purification, and elution, dATP overhangs were added to the DNA fragments. A paired-end P2 adapter containing T-overhangs was ligated to 20 μl of sheared, size-selected, P1-ligated, and pooled DNA templates with a specific barcode. The ligated material was then purified, eluted, and subjected to PCR enrichment and sequencing on a HiSeq2500 next-generation sequencing platform (Illumina, San Diego, CA, USA) with PE150 mode. Sequencing data for each individual were then de-multiplexed according to the specific barcode and index. Length of raw reads was 142–146 bp after removing barcode sequences. The original sequencing datasets have been deposited in the NCBI SRA repository with the accession number PRJNA516159.

### Read processing and SNP identification

Raw reads were processed with the *Stacks* pipeline (version 1.41) to call SNPs^57^. Raw reads were firstly filtered to generate clean reads with the *process_radtags* program (parameters: -r, -c, -q). Only the forward reads were kept for analysis as the reverse reads are at irregular distance from the restriction enzyme cutting site. To avoid artifacts, the following criteria were used to filter raw reads: (1) putative duplication reads generated by PCR amplification in library construction were discarded; (2) reads with adapter contamination were removed; (3) reads with ≥10% unidentified nucleotides (Ns) were discarded; (4) reads with average Pred quality score over a 15 bp sliding window below 10 (90% confidence) were removed; (5) reads without the correct partial 5 bp *EcoR*I recognition sequence (AATTC) were discarded. Then the *ustacks* program was used to align clean read sequences into exactly-matching stacks (or alleles) and compared the stacks to form a set of putative loci using a maximum likelihood framework for each individual (parameters: *-m* 3, *-M* 2). Loci that were two standard deviations above the mean depth of coverage were excluded, and then the *cstacks* program was used to merge loci of each parent into a catalog. The between-individual distance parameter of *cstacks* for mismatches was set 4 (parameter: *-n* 4). Then *sstacks* was run to match every individual in the population (including the two parents) against the catalog to identify locus/haplotype combinations in each individual. Finally, *genotypes* program was executed to call and export genotypes in a format compatible for *JoinMap* 4.0. The minimum number of matching progeny required for a locus was set as 122 to 140.

### Genetic linkage map construction

We used a two-step strategy to construct two versions of the map with different genotype profiles. The first version (Version 1.0) was constructed with markers that were genotyped in more than 94% of offspring (140 individuals, missing data rate <6%, strict parameter), while the second version (Version 2.0) was constructed with markers that were genotyped in more than 85% of offspring (122 individuals, missing data rate <18%, relaxed parameter). The genotype file with low data missing rate guaranteed the high quality of the map, whereas genotype file with high data missing rate was used to increase the marker density of the map. Genotype file of Version 1.0 was first imported into *JoinMap* 4.0^58^ by selecting the BC1 population type. Individuals with more than 30% missing markers were excluded. Markers showing severe Mendelian segregation distortion were also discarded (χ^2^ > 30, P < 0.01, d.f. = 2). Pairwise recombination estimates and a logarithm of odds (LOD) score of 6.0 were applied to determine linkage groups. Then single markers that were not assigned to any linkage group and linkage groups with less than three markers were excluded from further analysis. Markers in each LG were ordered using the regression algorithm with the parameters of a recombination rate of less than 0.4, a LOD value of greater than 1, and 3 rounds of ordering (a jump threshold of 5). The genetic distance (cM) was estimated with the *Kosambi* function^59^. After the initial mapping, double-crossovers were tested using the “*Genotype probabilities*” function. Suspicious genotypes were replaced with missing values and then re-ordered with the corrected genotyping matrix. Finally, distorted markers that did not affect the order of the surrounding markers were added to the linkage groups since these markers are known to barely affect the estimation of recombination frequency^26,60^. To validate the map quality, Version 1.0 map was analyzed with *Lep-Map* 2.0^61^. Only markers with orders consistent with that of *JoinMap* 4.0 were retained. *Lep-Map* 2.0 is capable of creating ultra-high-density linkage maps with high computational efficiency and accuracy implemented fully in JAVA. Because *JoinMap* 4.0 was unable to process the Version 2.0 genotyping data sets that contain larger set of markers, only *Lep-Map* 2.0 was adopted for grouping and ordering markers for this version. Finally, Version 2.0 map was compared to the Version 1.0 map, and markers with inconsistent orders were discarded. The final map was drawn with *MapChart* 2.2^62^.

To evaluate the map quality, heat map reflecting the recombination relationship between markers in each LG was generated with *CheckMatrix*^63^, and haplotype map mirroring the double crossover were generated by *JoinMap* 4.0 for each LG respectively (for Version 1.0 map only). A 6-kb sequencing library was constructed and sequenced in paired-ends to evaluate the map quality by Blast searching for neighboring markers using *ustacks* program (Supplemental Fig. S8). The coding of linkage groups and their corresponding relationships between Version 1.0 and Version 2.0 is shown in Supplementary Table S3. Genome coverage of the newly-built linkage map was estimated with the equation *c* = 1-*e*^−*2dn/L*^, where *d* is the average interval of markers, *n* is the number of markers, and *L* is the length of the linkage map estimated by *L* = l*(m + *1)/(m* − *1)* (*m* is the number of markers in the linkage group)^64^.

### Anchoring contigs from the rose genome assemblies to the linkage map

The OB genome was recently obtained from doubled-haploid plants using single-molecule real-time sequencing^6,10^. To verify the collinearity between our genetic map and the reference genomes and to place more contigs to the pseudo-chromosomes, we anchored the contigs to the linkage map using RAD-tag markers. Initially, all markers from the high density genetic map were searched on the genome assembly with *BLAST* + *2.6.0* to integrate contig sequences^65^. Full genome sequence was downloaded from Genome Database for Rosaceae (https://www.rosaceae.org). The marker sequences were blast searched against contigs with a cutoff E-value of 10^−15^. If a query hit one contig, the contig was assigned to the LG; if a query hit two or more contigs with a less than two-fold difference in the E-value, we did not assign any of these contigs. In cases where a contig was hit by multiple markers from different linkage groups, the contig was assigned to LGs with more than 2/3 markers. Inconsistency between the LGs and the pseudo-molecules of the assemblies was used to detect chromosome rearrangement events. Finally, total length of contigs anchored to the LGs was calculated. Collinearity information was generated using *Circos* software^66^.

To identify centromeric contigs, we aligned the 13 centromeric repeat sequences identified by Raymond *et al*.^6^ using Blast^67^. Gene and transposable element (TE) annotations were retrieved for both genome assemblies. We filtered out TEs classified as “putative host genes”, and discarded gene annotations having their exons overlapped by TE annotations on more than 30% of their length. We also identified chloroplastic and mitochondrial contigs from Hibrand Saint-Oyant’s genome by comparing them to reference sequences CM009590.1, KF753637.1, NC_037492.1, NC_032038.1, CM009589.1 and NC_018554.1. For all these features and Blast results, we computed the percentage of the sequence length they were covering (Supplementary Tables S8 and S9).

### Sequence comparison with *Fragaria vesca*

To examine the conservation of synteny between rose and strawberry (*F. vesca*), the marker sequences of our genetic map were searched against the genome sequences of *Fragaria* (v1.1 and v4.0.a1 assemblies; http://www.rosaceae.org) using the *BLAST* + *2.6.0* program with default parameters^65^. Markers with low quality ratio were excluded: (1) The length of the alignment was less than 100 bp; (2) The hit was found at multiple locations on the scaffolds (potentially corresponding to repetitive elements); (3) E-value < 10^−5^. Alignments between rose LGs and the strawberry genome were visualized with *Circos*^66^.

## Supplementary information

supplementary figures 1-10

supplementary tables 1-10

## Data Availability

All data supporting the results of this study are included in the manuscript and its additional files.
