# Supplementary material for: The development of a high-density genetic map significantly improves the quality of reference genome assemblies for rose
Source: Sci Rep. 2019 Apr 12;9:5985. doi: 10.1038/s41598-019-42428-y (PMC6461668; doi:10.1038/s41598-019-42428-y)
Supplement: Supplementary file 1 — supplementary figures 1-10 [file 41598_2019_42428_MOESM1_ESM.pdf]

The development of a high-density genetic map significantly improves the quality of reference genome assemblies for rose

Shubin Li [1,#], Guoqian Yang [2,#], Shuhua Yang [3,#], Jeremy Just [4], Huijun Yan [1], Ningning Zhou [1], Hongying Jian [1], Qigang Wang [1], Min Chen [1], Xianqin Qiu [1], Hao Zhang [1], Xue Dong [2], Xiaodong Jiang [2,6], Yibo Sun [2,6], Micai Zhong [2,6], Mohammed Bendahmane [4], Guogui Ning [5], Hong Ge [3,*], Jin-Yong Hu [2,*], Kaixue Tang[1,*]

1, National Engineering Research Center For Ornamental Horticulture; Flower Research Institute, Yunnan Academy of Agricultural Sciences; Yunnan Flower Breeding Key Lab, Kunming 650231, China.

2, CAS Key Laboratory for Plant Diversity and Biogeography of East Asia, Kunming Institute of Botany, Chinese Academy of Sciences. Kunming 650201, China.

3, Institute of Vegetables and Flowers, Chinese Academy of Agricultural Sciences, Beijing 100081, China.

4, Laboratoire Reproduction et Développement des Plantes, Univ Lyon, ENS de Lyon, UCB Lyon 1, CNRS, INRA, F-69364 Lyon, France.

5, Key laboratory of Horticultural Plant Biology, Ministry of Education, College of Horticulture & Forestry Sciences, Huazhong Agricultural University, Wuhan 430070, China.

6, Kunming College of Life Sciences, University of Chinese Academy of Sciences, Kunming 650201, Yunnan Province, China.

[#], These authors contributed equally;

[*], Authors for correspondence: kxtang@hotmail.com; hujinyong@mail.kib.ac.cn; or gehong@caas.cn.

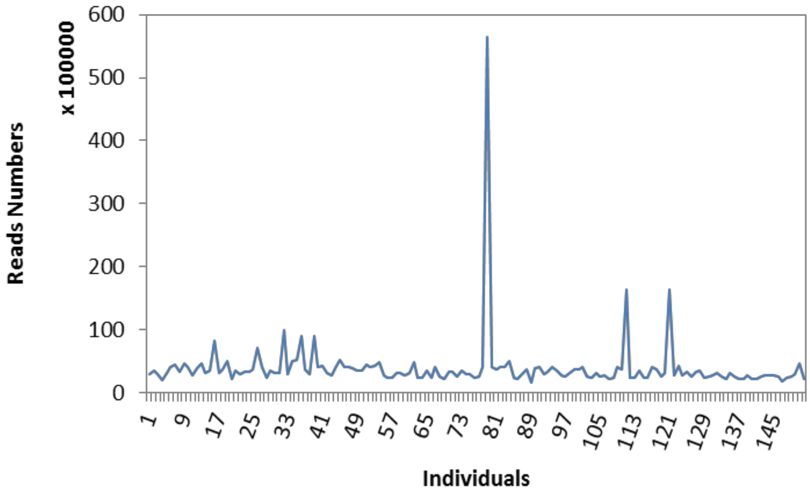

Figure S1. Line chart showing genome sequencing data of the *Rosa chinensis* 'Old Blush' (OB) and *R. wichuriana* 'Bayes' Thornless' (BT) population.

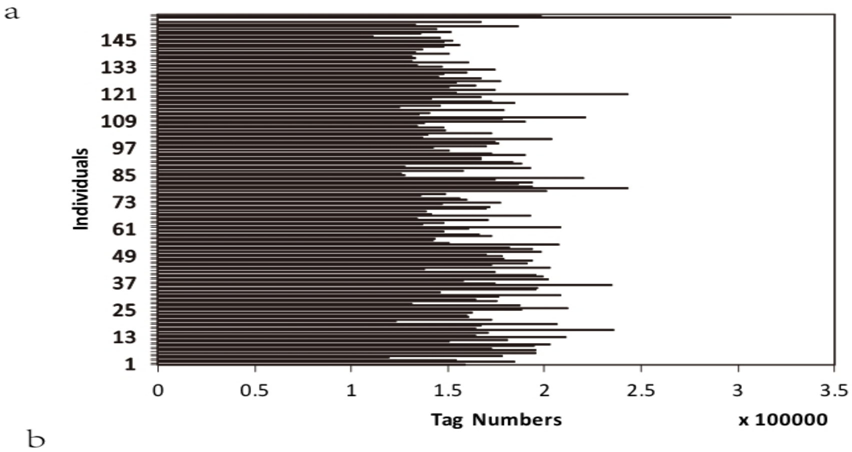

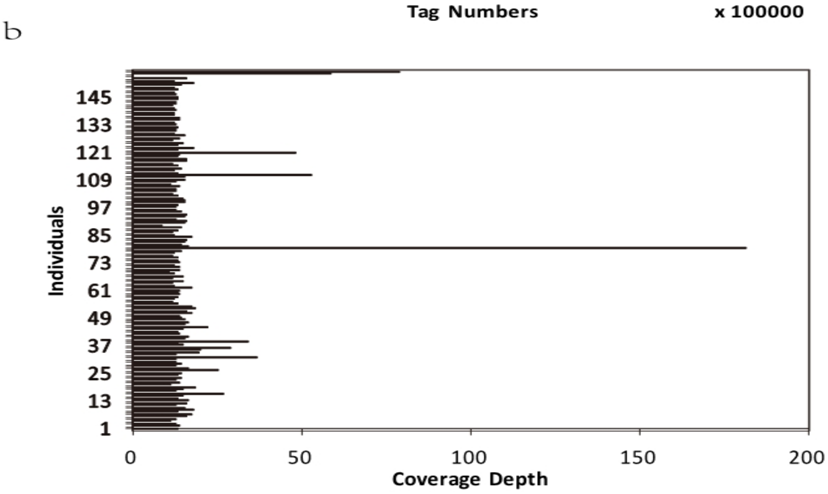

Figure S2. Number of markers and average sequencing depth for each of the BC1 individual and their parents. The y-axes in a and b indicate the plant accession including the female parent and the male parent followed by each of the BC1 individuals, the x-axes indicates number of tags in a and coverage in b.

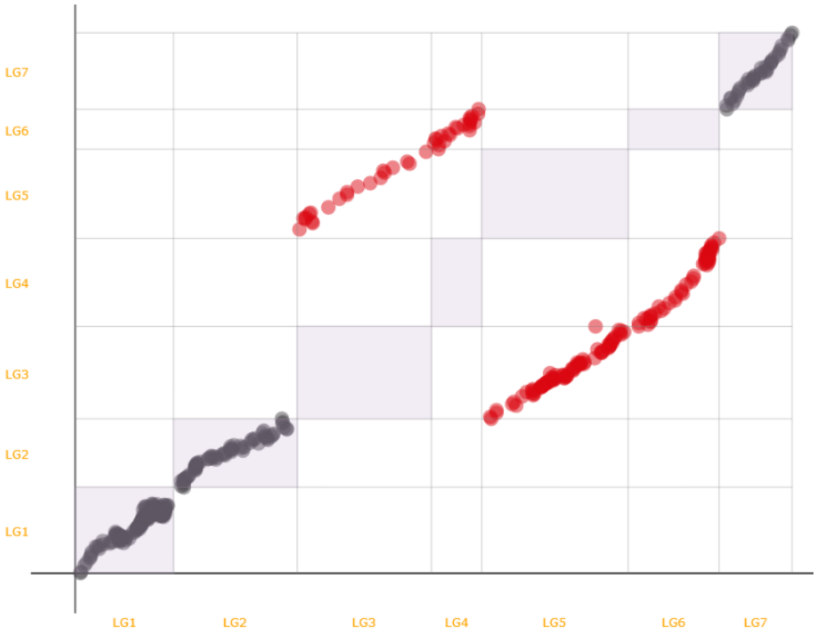

Figure S3. Correlation of the Version 1.0 (Y axis) and Version 2.0 (X axis) maps.

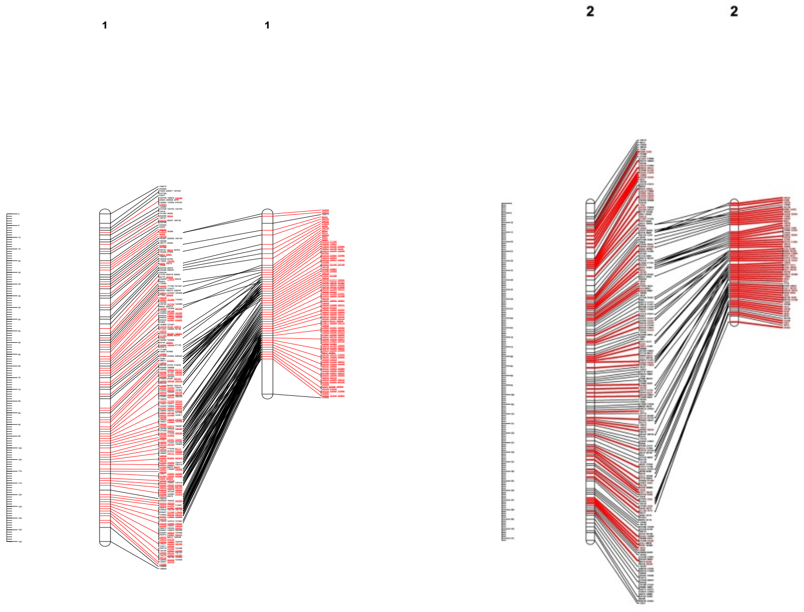

Figure S4. Synteny analysis between the Version 1.0 and Version 2.0 maps. Continued-

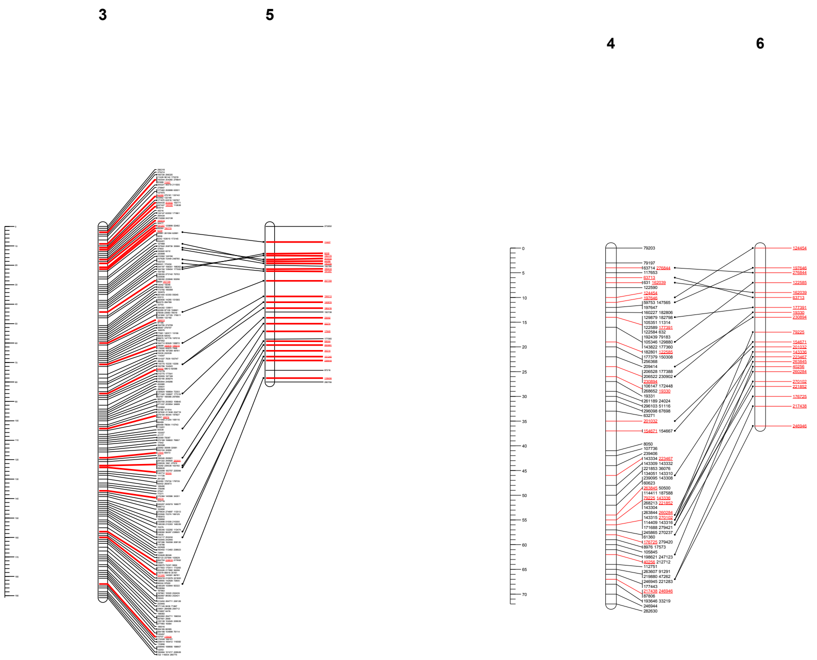

Figure S4. Synteny analysis between the Version 1.0 and Version 2.0 maps. Continued-

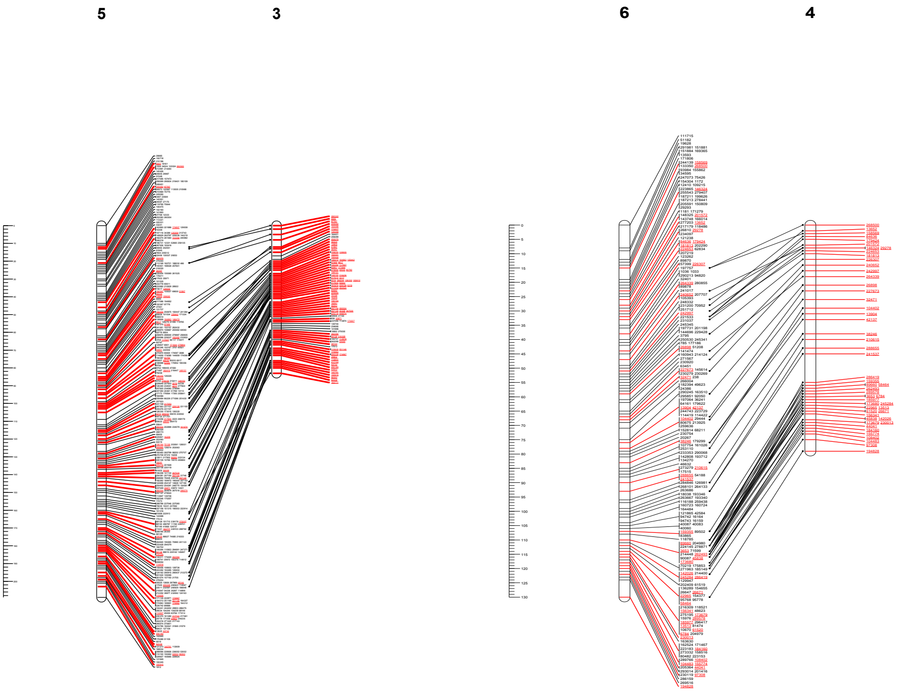

Figure S4. Synteny analysis between the Version 1.0 and Version 2.0 maps. Continued-

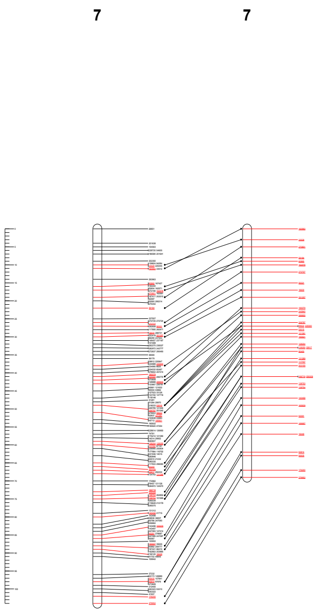

Figure S4. Synteny analysis between the Version 1.0 and Version 2.0 maps.

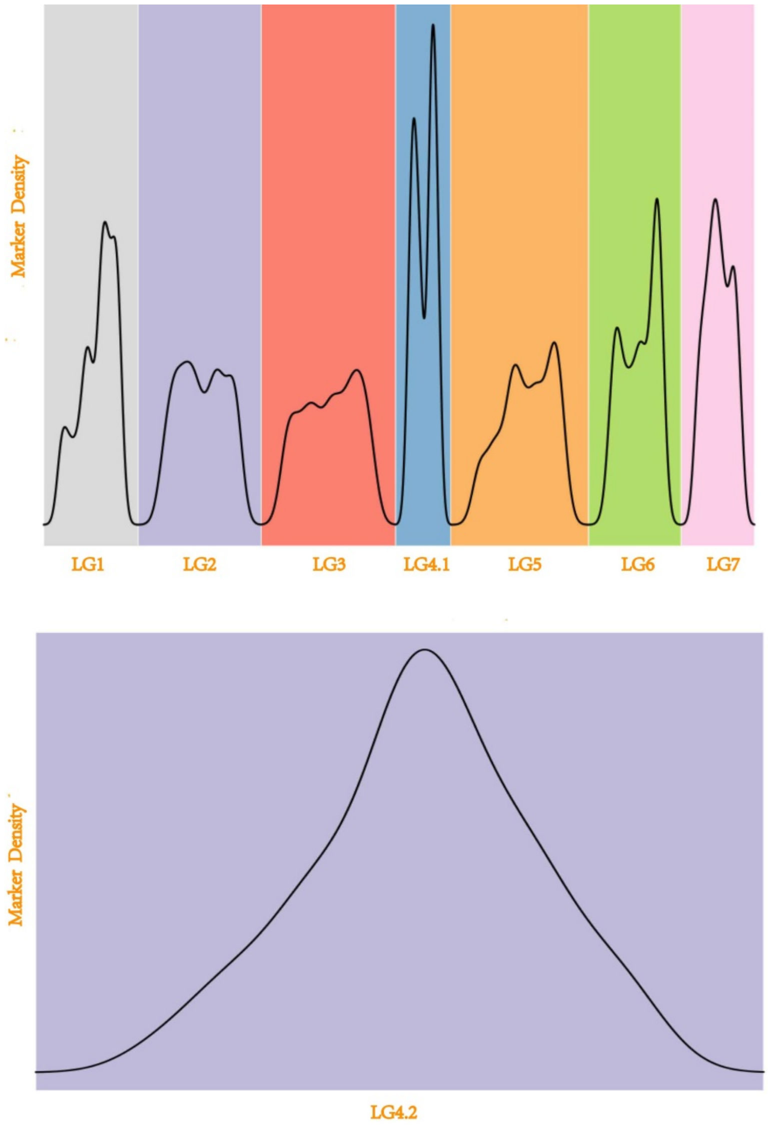

Figure S5. Marker distribution and density along chromosomes. Chromosome length variations among LGs and regions with unusually high/low genetic marker density are shown.

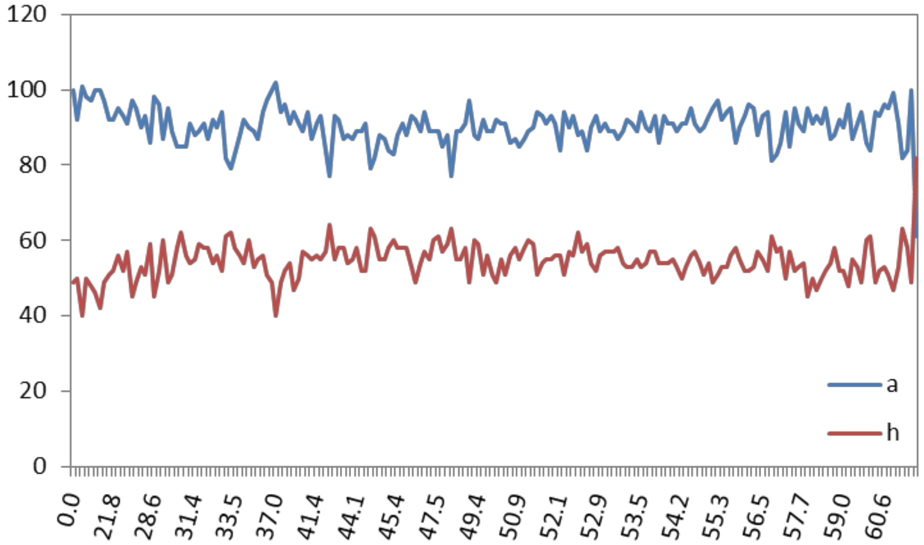

Figure S6a. Number of homozygotes and heterozygotes on each LG of Version 2.0 map. X axis indicates the genetic distance of LG1, while the y-axis indicates number of individuals in the BC1 plant accessions. Continued-

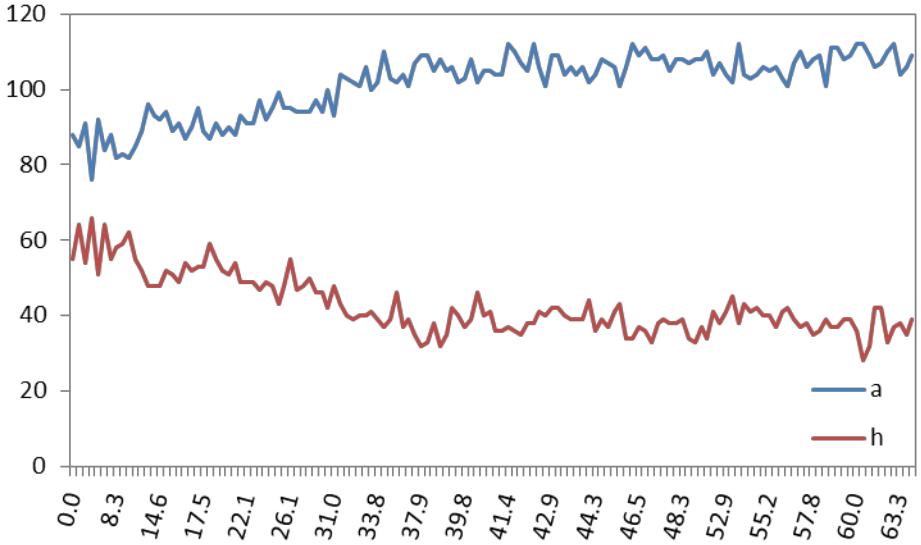

Figure S6b. Number of homozygotes and heterozygotes on each LG of Version 2.0 map. X axis indicates the genetic distance of LG2, while the y-axis indicates number of individuals in the BC1 plant accessions. Continued-

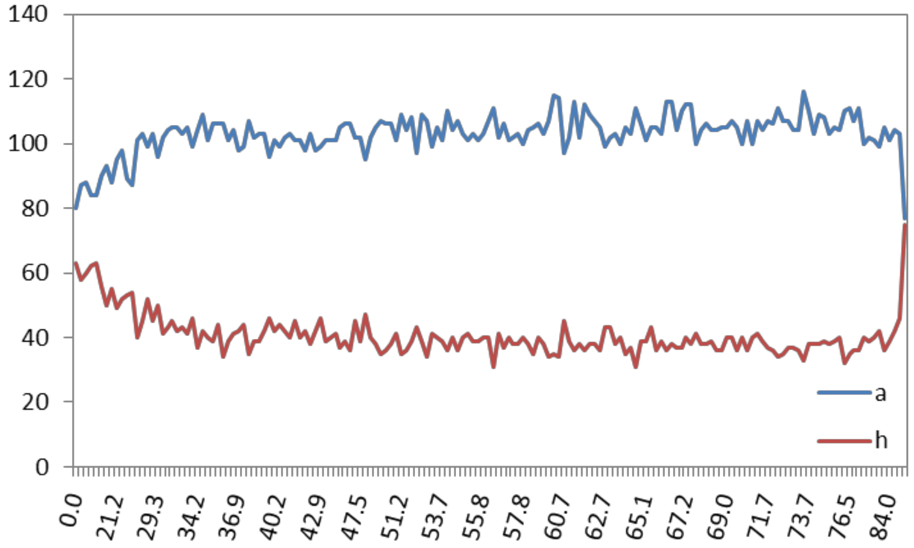

Figure S6c. Number of homozygotes and heterozygotes on each LG of Version 2.0 map. X axis indicates the genetic distance of LG3, while the y-axis indicates number of individuals in the BC1 plant accessions. Continued-

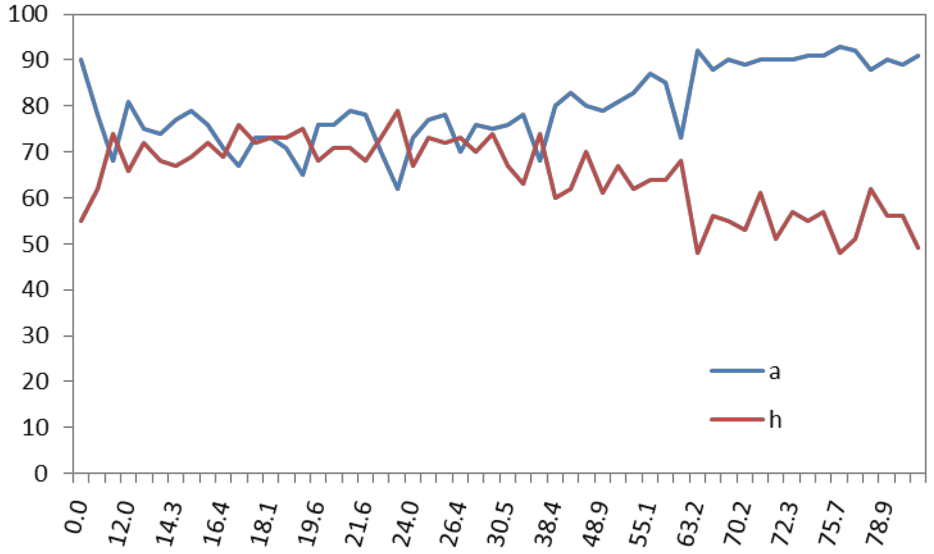

Figure S6d. Number of homozygotes and heterozygotes on each LG of Version 2.0 map. X axis indicates the genetic distance of LG4, while the y-axis indicates number of individuals in the BC1 plant accessions. Continued-

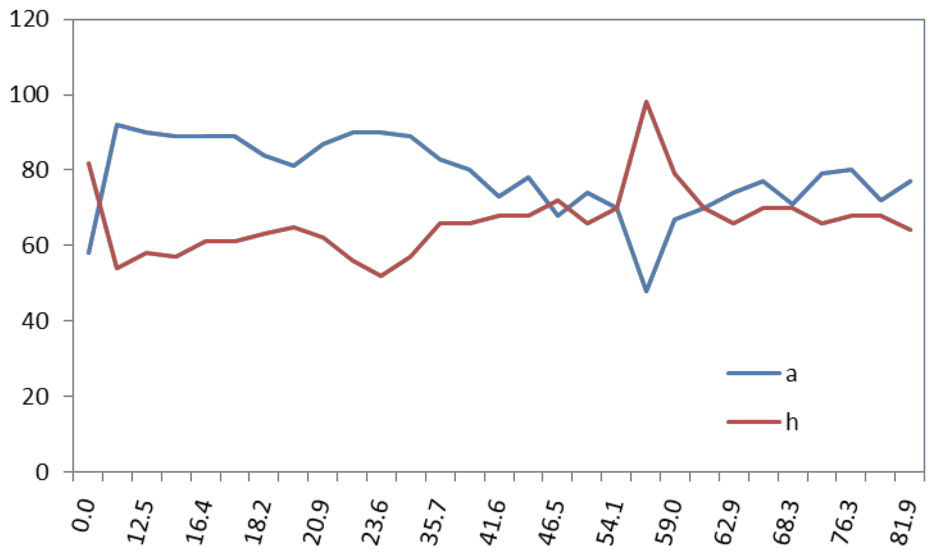

Figure S6e. Number of homozygotes and heterozygotes on each LG of Version 2.0 map. X axis indicates the genetic distance of LG5, while the y-axis indicates number of individuals in the BC1 plant accessions. Continued-

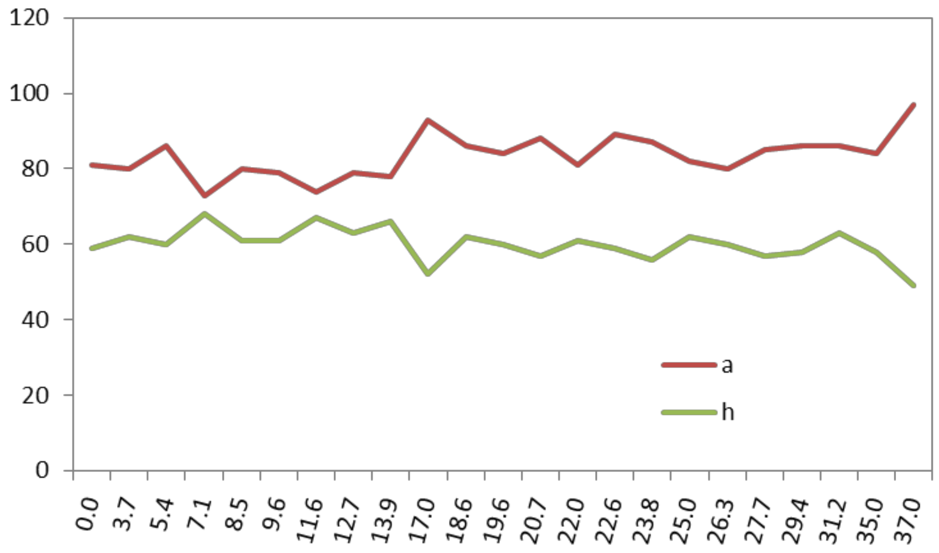

Figure S6f. Number of homozygotes and heterozygotes on each LG of Version 2.0 map. X axis indicates the genetic distance of LG6.1, while the y-axis indicates number of individuals in the BC1 plant accessions. Continued-

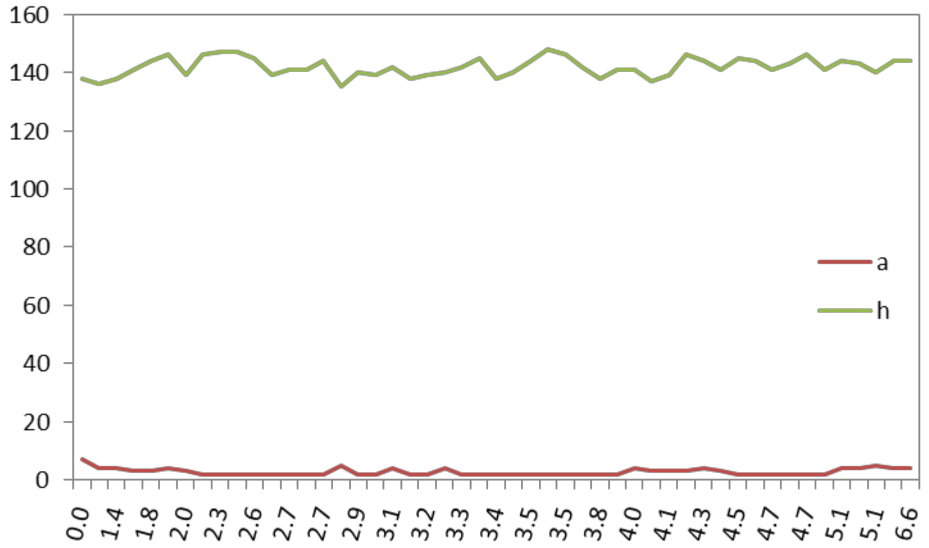

Figure S6g. Number of homozygotes and heterozygotes on each LG of Version 2.0 map. X axis indicates the genetic distance of LG6.2, while the y-axis indicates number of individuals in the BC1 plant accessions. Continued-

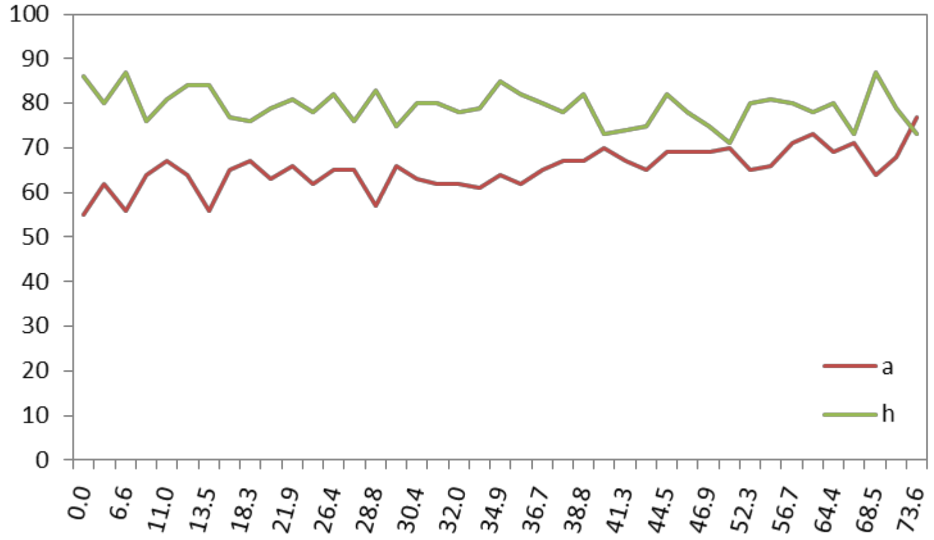

Figure S6h. Number of homozygotes and heterozygotes on each LG of Version 2.0 map. X axis indicates the genetic distance of LG7, while the y-axis indicates number of individuals in the BC1 plant accessions. Continued-

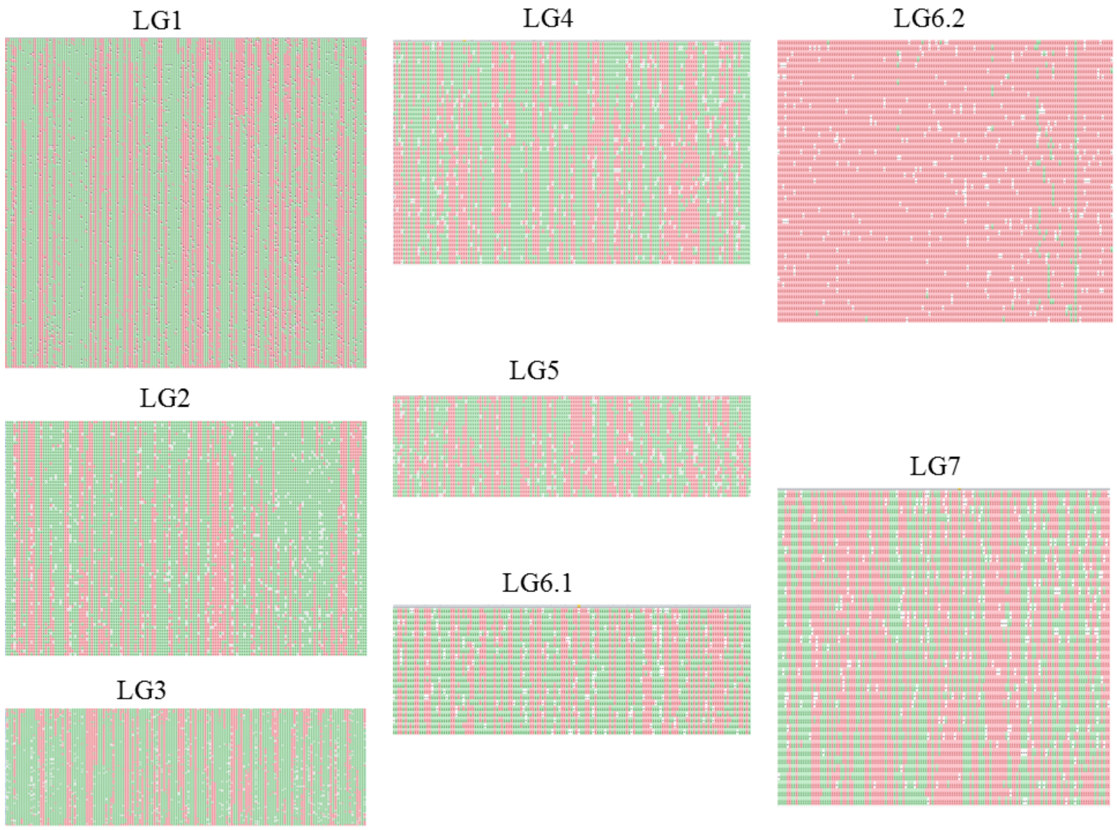

Figure S7. Haplotype map of the Version 1.0 genetic map. Green represents homozygotes, red indicates heterozygosity and white represents missing data. Each column represents the genotype of an individual in the BC1F1 population. Rows correspond to individual genetic marker.

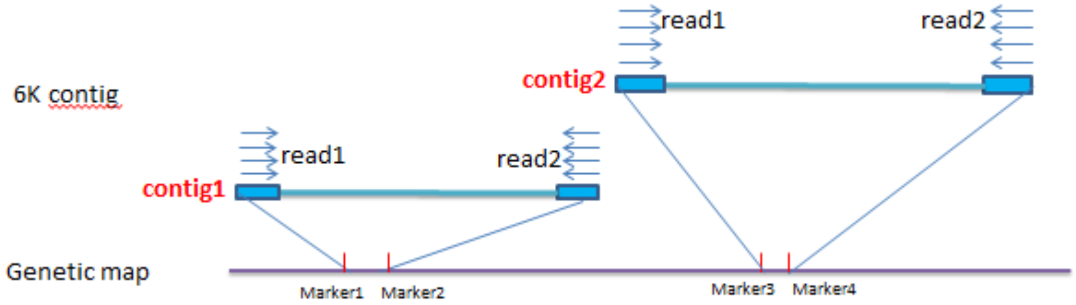

Figure S8. Schematic diagram showing genetic markers alignment with 6K library contigs. We first generate a long read by connecting the read1 and read2 end to end. Then similar long reads were clustered into contigs using *ustacks* (V1.41) software. Finally markers were blast searched on the contigs (blastn, E^{-10}).

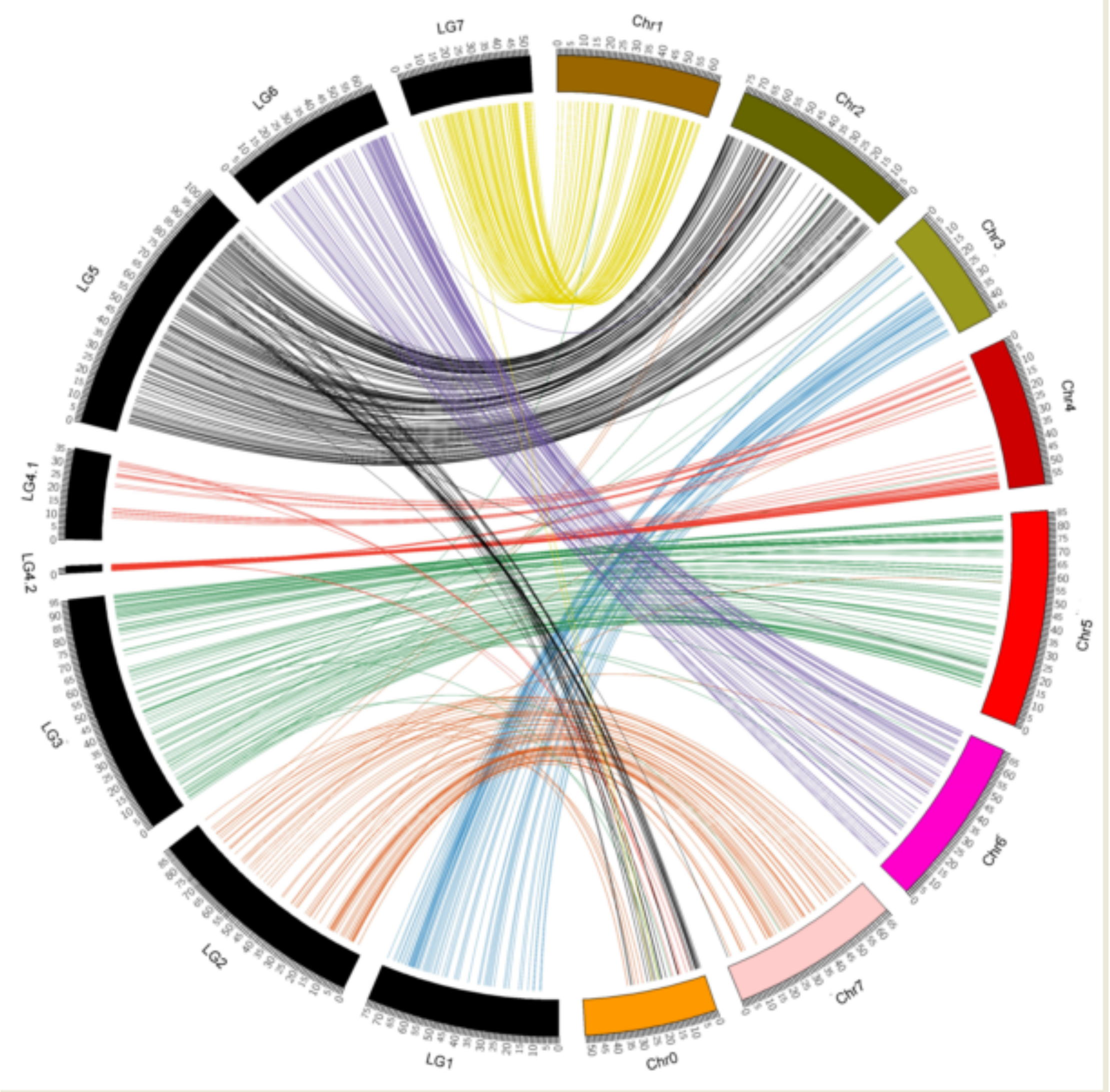

Figure S9. Conservation of synteny between the constructed genetic map (Version 2.0) and the OB reference genome (ref.10). LGs 1-7 indicate linkage groups in this research while Chromosomes 0-7 represent the published OB chromosomes (ref. 11).

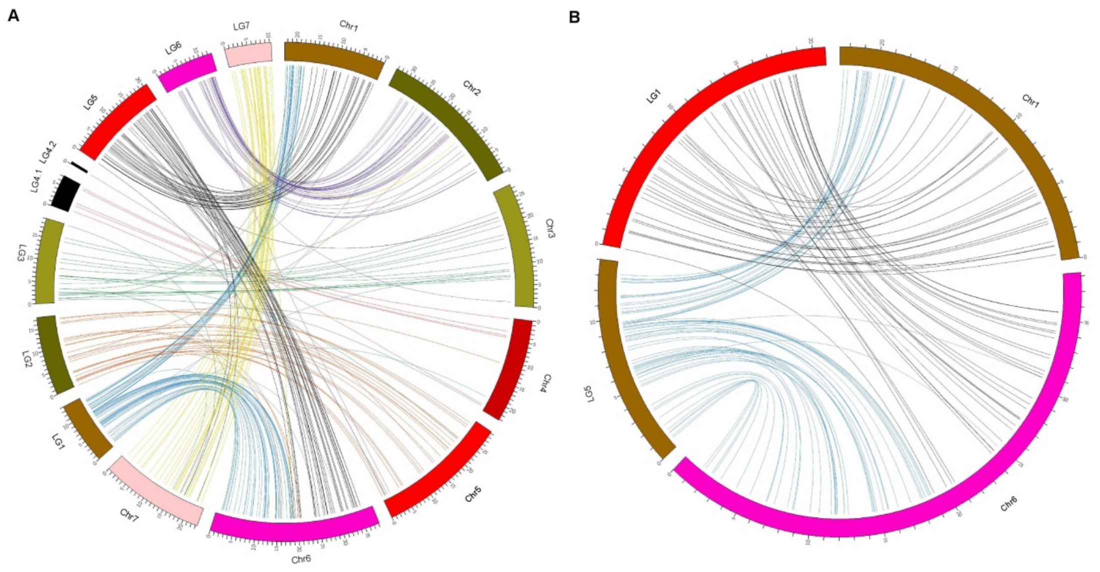

Figure S10. Conservation of synteny between the constructed linkage map (Version 2.0) and the *Fragaria vesca* (v4.0.a1) genome (ref. 49).

a. Comparison of the synteny between the constructed LGs and the seven *F. vesca* Chromosomes.

b. Detailed comparison of the syntenic relationship between LG1 and LG5 of our new genetic map and *F. vesca* Chr1 and Chr6. The newly constructed LGs are in the left part, while *F. vesca* chromosomes are on the right part of the circles.
